# An adolescent girl with signs and symptoms of anaphylaxis and negative immunologic workup: a case report

**DOI:** 10.1186/s13256-020-02371-3

**Published:** 2020-04-17

**Authors:** Sarah Green, Allison Hicks, Chelsea Hilsendager, Maureen Bauer, Guido K. W. Frank

**Affiliations:** 1 Department of Pediatrics, Section of Adolescent Medicine, Children’s Hospital Colorado, 13123 E. 16th Ave, Box 025, Aurora, CO 80045 USA; 2 Department of Pediatrics, University of Colorado School of Medicine, Aurora, CO USA; 3 Department of Pediatrics, Section of Allergy and Immunology, Children’s Hospital Colorado, Aurora, CO USA; 4 Department of Psychiatry, Division of Child and Adolescent Psychiatry, University of Colorado Anschutz Medical Campus, Aurora, CO USA; 5 Department of Psychiatry, University of California San Diego, San Diego, CA 92121 USA

**Keywords:** Anaphylaxis, Angioedema, Somatic, Eating disorder, Health care costs

## Abstract

**Background:**

The increasing incidence of allergies and allergic reactions among children and adults has become a major public health concern. The etiology of allergic reactions can often be confirmed based on a detailed history and supportive testing. However, there are cases where the underlying factors are more complex and difficult to identify.

**Case presentation:**

Here we present the case report of a 14-year-old Caucasian  girl with weight loss and a 3-year history of reported angioedema culminating in five intensive care unit admissions over the course of 2.5 months. Her initial clinical presentation included hypotension, dyspnea, and reported facial edema, but allergy and immunological workup were negative. A psychiatric workup identified an eating disorder with food restriction, comorbid major depression, obsessive compulsive disorder, and posttraumatic stress disorder. A close collaboration between Adolescent Medicine, Allergy and Immunology, and Psychiatry helped disentangle medical from psychiatric problems, including fluoxetine medication effects, as well as develop a treatment plan that was acceptable to the family. The angioedema was ultimately diagnosed as factitious disorder.

**Conclusions:**

This patient’s treatment highlights the importance of a multidisciplinary team approach, a multifactorial etiology that needed to be addressed by multiple specialists, and the importance of long-term treatment and support.

## Background

The differential diagnosis for allergic reactions is wide and can include idiopathic anaphylaxis, IgE-mediated reactions, mastocytosis, hereditary angioedema, and vocal cord dysfunction (VCD). In patients with eating disorders, aversion to food can further complicate this differential. Medications can add increasing complexity to the clinical picture. For example, case reports have cited angioedema correlated with increasing doses of fluoxetine [1, 2].

This case report examines the clinical presentation and treatment course of a patient with episodes that were initially diagnosed as anaphylaxis. The aim of this report is to discuss possible etiologies of her condition and highlight the importance of maintaining a differential diagnosis, especially for a patient with a potentially fatal condition.

## Case presentation

A 14-year-old Caucasian girl was transferred from a community hospital emergency department (ED) where she was treated for symptoms concerning for anaphylactic reaction to a hypoallergenic nutritional supplement. Immediately after ingestion she reported wheezing, cough, and swelling of lips, face, and tongue. She had administered epinephrine 0.3 mg at home.

She had a history of suspected food allergies, intermittent asthma, complex regional pain syndrome, and delayed gastric emptying. Her reported allergies included wheat, oats, tree nuts, chocolate, eggs, cow’s milk, and rice. However, it was unclear as to what her prior reactions had been to these stimuli. Skin prick testing and serum-specific immunoglobulin E (IgE) to foods of concern were negative, other than sensitization to several tree nuts without a supportive history of reaction with ingestion. Her parents had declined observed food challenges, the gold standard for food allergy diagnosis. She had negative testing for hereditary angioedema as well as normal baseline tryptase, an acute indicator of mast cell degranulation which is typically elevated in mast cell disorders and often, but not always, acutely elevated in anaphylaxis.

She was living with her mother and father. She was in ninth grade, being homeschooled after leaving public school secondary to fears about her allergies. She was up to date on vaccinations. Medications prior to admission included albuterol two puffs as needed, dicyclomine 10 mg as needed, montelukast 10 mg daily, fluoxetine 60 mg daily, diphenhydramine 50 mg as needed, and epinephrine 0.3 mg as needed.

She had lost 18 kg (40 lbs) in the year prior to admission due to food restriction and exercise. Her body mass index (BMI) was at the 63rd percentile, down from the 94th. Stressors included moving across the country and bullying victimization. She became increasingly anxious and depressed in her new environment. She was started on fluoxetine for major depression, and up-titrated to 60 mg per day 5 months earlier.

### Clinical course in the ED

She had increased work of breathing on arrival with poor air movement, stridor, and biphasic audible wheezing. Her lips were pursed, but she had no swelling of lips, tongue, or oropharynx. She had no deficits on neuro examination. On initial evaluation she was afebrile with blood pressure of 94/48, pulse of 100, respiratory rate of 30, and blood oxygen saturation (SpO_2_) of 100%. Her minimum SpO_2_ was 96%, and respiratory rate in the ED was 20–66. She received two boluses of normal saline 1000 mL, two doses of epinephrine 300 mcg, albuterol 2.5 mg every 15 minutes, ipratropium-albuterol 0.5–3 mg, and 0.5 mL racemic epinephrine 2.25%. She was then admitted to the pediatric intensive care unit (PICU). Her angioedema resolved after two total doses of subcutaneous epinephrine and six doses of intravenously administered diphenhydramine. She was then transferred to a tertiary care eating disorder treatment center.

### Admission to the eating disorder program

She was diagnosed as having other specified eating disorder (OSFED) anorexia nervosa type (meeting anorexia nervosa criteria except the low weight criterion), major depressive disorder (MDD), social anxiety disorder, obsessive compulsive disorder (OCD), and posttraumatic stress disorder (PTSD) secondary to bullying. She reported several suicide attempts when severely depressed, which had been the reason for gradual increase of fluoxetine.

While in the eating disorder program, she had seven episodes concerning for idiopathic anaphylaxis requiring transfer to the ED, four of which resulted in PICU admissions. She was treated with intramuscular epinephrine, antihistamines, and steroids. Four episodes occurred in response to foods to which she had no known allergy, two occurred just after transfer from the PICU to medical floors, and one upon smelling perfume. She usually complained about perioral swelling and difficulty breathing. Providers had difficulty confirming visible swelling. She never had an urticarial rash or protracted vomiting. Once, she had inspiratory vocalizations which paused with coughing.

She had low blood pressure at baseline, and during three of her ED visits she had hypotension, ranging from 72/45 to 109/41, resulting in initiation of intravenously administered epinephrine or norepinephrine in the PICU. She did not have oxygen desaturations. She was electively intubated once for suspected airway angioedema due to persistence of stridor despite intramuscular epinephrine; however, upon intubation the airway was patent without evidence of inflammation. During another episode, direct laryngoscopy performed 1 minute after stridor resolved showed no airway edema or VCD (see Table 1 for full workup).

**Table 1:** Patient's workup throughout her hospitalization

Test	Result
Direct laryngoscopy	No paradoxical vocal cord movement or dysfunction
Complement	Normal C4, C1esterase inhibitor level and function
Skin prick test	Sensitization to hazelnut, pecan, and walnut, otherwise negative
Serum specific IgE	< 0.35 kU/L (normal) for foods of concern except for several tree nuts per outside allergist’s report
Serum tryptase	2.4, 1.2, 1.6, 1.7, and 3.8 ng/ml (normal < 11.5 mg/ml)
Bone marrow biopsy	Equivocal
Urine N-methylhistamine	203 and 394 mcg/g (normal range 70–333 mcg/g)
Urine prostaglandins (PG D2)	70 ng/24 hours (normal range 100–280 ng/24 hours)
Urine 2,3-dinor 11B-prostaglandin F2a	1333 pg/mg (normal level < 5205 pg/mg in adults, no pediatric reference range available)
Complete blood count	Within normal limits
Comprehensive metabolic panel	Within normal limits

To confirm anaphylaxis, she had five serum tryptase levels drawn during acute episodes, but all had negative results. Mast cell activation syndrome was ruled out based on no significant increase in serum tryptase or other mast cell mediators when symptomatic. A bone marrow biopsy to assess for a mast cell disorder was equivocal due to poor sample. She was prescribed fexofenadine 180 mg daily, ranitidine 150 mg twice daily, cromolyn 200 mg, and a prolonged prednisone course, weaned down slowly from 30 mg. Rescue medications included cetirizine 10–20 mg and lorazepam 1–2 mg given the concern that episodes were at least in part due to anxiety. Diphenhydramine was avoided due to prolonged QTc during one PICU admission.

She self-harmed and had intermittent suicidal ideation and auditory hallucinations consistent with major depressive illness. She was started on aripiprazole and up-titrated to 10 mg, which stopped the hallucinations and improved depression. A chart review indicated that the reported angioedema episodes had increased in frequency and severity with each fluoxetine increase. She was weaned off fluoxetine, and angioedema attacks decreased. When discharges were planned, she repeatedly became suicidal or reported another angioedema episode. She was observed to be triggering angioedema attacks by secretly eating food (for example cookies with nuts) that she was told not to eat because of potential anaphylactic reaction. She identified a fear of going home and a need for attention. She felt her brother with a developmental disorder was receiving most of the attention from her parents. Borderline personality disorder was considered but ruled out as she did not have avoidance of abandonment, unstable intense relationships, self-damaging impulsivity, intense anger, or feelings of emptiness outside severe depression. Psychotherapy focused on the hypothesis that she was assuming the sick role to receive attention, which successfully helped balance the family structure.

She was discharged after 15 weeks. Her final diagnoses, in addition to the psychiatric diagnoses mentioned above, included: possible but unconfirmed idiopathic angioedema, possible VCD, and factitious disorder. At the time of this writing, she had one ED visit for angioedema 2 months after discharge, but had not been admitted to a PICU in 22 months.

## Discussion and conclusions

This case involves a 14-year-old girl who had multiple episodes concerning for anaphylaxis, which presented with perioral edema and dyspnea. While her workup for anaphylaxis was negative, she was found to have OSFED, MDD, social anxiety disorder, OCD, and PTSD. This patient was treated for anaphylaxis without meeting National Institute of Allergy and Infectious Diseases (NIAID) criteria on several occasions [3, 4] and she never had objective findings of anaphylaxis. The hospitalization incurred a bill of US$243,000. Earlier exclusion of anaphylaxis could have reduced this expense.

Few other cases of factitious angioedema have been reported in the literature. This case is unusual as our patient was an adolescent and presented with a comorbid eating disorder. Both Feldman et al*.* [5] and Choy et al. [6] have described adult patients with similar presentations and negative workup. Their patients were all referred to psychiatric care, although many patients did not adhere to recommendations, so conclusions cannot yet be made regarding treatment efficacy.

Our patient posed a diagnostic dilemma. She presented with prior history of food allergy, which created increased anxiety for her and for her providers, although skin prick and serum-specific IgE tests had been negative. Several differential diagnoses were considered, including idiopathic anaphylaxis, IgE-mediated reactions, mastocytosis, hereditary angioedema, and VCD. Normal tryptase levels and lack of adequate response to epinephrine made an anaphylactic reaction less likely. However, a prolonged steroid taper for treatment of frequent idiopathic anaphylaxis was prescribed prophylactically [7, 8].

No VCD was seen in the acute setting; however, laryngoscopy is not highly sensitive [9, 10] and repeat laryngoscopy after discharge was consistent with VCD. She has constitutionally low blood pressure, which predisposed her to severe hypotension and may have mimicked hypotensive episodes. The hypotension noted during her episodes was in the range of her baseline blood pressure. The possibility of angioedema could not be ruled out and could have been exacerbated by increasing doses of fluoxetine, which has been cited in case reports [1, 2]. She may have learned that her medical condition could provide her with parental attention. At the time, she did not provide diagnostic symptoms for borderline personality disorder, but later development cannot be ruled out. It seems she presented with self-induced stridor and subjective versus true angioedema [5]. We diagnosed her as having factitious disorder, characterized by falsification of medical or psychological symptoms to receive treatment.

This case exemplifies how non-resolving medical problems may be complex interactions of somatic-medical pathologies with psychiatric illness. It was critical to examine the interaction of her physical symptoms with her psychiatric diagnoses. For example, the fear of eating likely exacerbated the reactions that occurred during meals. The cooperation of a multidisciplinary team was key to resolving her condition and helping the family to develop a psychosomatic conceptualization of her problems.

## Data Availability

Not applicable.

## Abbreviations

BMI: Body mass index
ED: Emergency department
MDD: Major depressive disorder
NIAID: National Institute of Allergy and Infectious Diseases
OCD: Obsessive compulsive disorder
OSFED: Other specified eating disorder
PICU: Pediatric intensive care unit
PTSD: Posttraumatic stress disorder
SpO_2_: Blood oxygen saturation
VCD: Vocal cord dysfunction
